# The impact of low carbohydrate diet on the plasma proteomic profile of individuals with obesity

**DOI:** 10.3389/fnut.2026.1856332

**Published:** 2026-09-09

**Authors:** Nora A. Alfadda, Ghadeer S. Aljuraiban, Salini Scaria Joy, Heba Elkhateb, Shahed Nawaz, Othman Mahmoud Othman, Osama Z. Alzeer, Abdullah Abdulaziz Al Wabel, Assim A. Alfadda, Hicham Benabdelkamel

**Affiliations:** 1 Department of Community Health Sciences, Clinical Nutrition, College of Applied Medical Sciences, King Saud University, Riyadh, Saudi Arabia; 2 Strategic Center for Diabetes Research, College of Medicine, King Saud University, Riyadh, Saudi Arabia; 3 Proteomics Resource Unit, Obesity Research Center, College of Medicine, King Saud University, Riyadh, Saudi Arabia; 4 Department of Clinical Nutrition, King Khalid University Hospital, King Saud University, Riyadh, Saudi Arabia; 5 University Diabetes Center, King Saud University, Riyadh, Saudi Arabia; 6 Department of Medicine, College of Medicine, King Saud University, Riyadh, Saudi Arabia

**Keywords:** dietary intervention, low carbohydrate diet, obesity, omics, plasma proteomics, weight loss

## Abstract

**Introduction:**

Obesity is a major public health concern worldwide and contributes to multiple metabolic disorders. Low carbohydrate diets (LCDs) have been proposed as an effective strategy for weight management and metabolic improvement; however, the mechanisms underlying LCD-induced molecular changes in individuals with obesity remain incompletely understood. This study aimed to assess the impact of a three-month LCD on the proteomic profile of adults with obesity.

**Methods:**

Thirty adults (18–50 years) with obesity (BMI ≥ 30 kg/m^2^) followed an LCD (60–110 g/day) for 3 months. Anthropometric and laboratory measurements were obtained, and blood samples were collected at baseline and endpoint. Changes in the plasma proteomic profile were evaluated using an untargeted label-free quantitative proteomics approach (LC–MS/MS), complemented by bioinformatics and pathway network analyses. Anthropometric and biochemical data were analyzed using paired-sample *t*-tests and Wilcoxon signed-rank tests in IBM SPSS®.

**Results:**

Overall, the LCD was associated with significant reductions in body weight, BMI, waist and hip circumferences, fat mass and percentage, GGT, triglycerides, and HbA1c (paired *t*-test, *p* < 0.001). Participants were stratified by total weight loss percentage into responders (≥5%; *n* = 15) and non-responders (<5%; *n* = 15). Label-free proteomics identified 90 significantly dysregulated proteins in responders (53 upregulated, 37 downregulated) and 183 in non-responders (111 upregulated, 72 downregulated) between baseline and endpoint. Significantly dysregulated proteins in the responder group included beta-enolase (ENO3) and CREG1 (both upregulated), and tenascin (downregulated). In the non-responder group, insulin-like growth factor-binding protein 7, acyl-CoA-binding protein, and talin-1 were significantly downregulated. Pathway network analysis in responders was centered around dysregulation of TNF, IL6, IFNG, APP, DEGS1, and ENO3 and was related to cell-mediated immune response, cell-to-cell signaling and interaction, and cellular movement. In non-responders, the top network was centered around PGAM1, PDIA3, PHGDH, and EGFR and was associated with cardiovascular disease, dermatological diseases and conditions, and organismal injury and abnormalities.

**Conclusion:**

A three-month LCD significantly improved anthropometric and metabolic parameters in adults with obesity and was associated with marked changes in the plasma proteome. Distinct proteomic signatures between responders and non-responders highlight inter-individual variability in biological response, supporting the potential of plasma proteomics for biomarker discovery and precision nutrition in obesity management.

## 1 Introduction

Obesity has emerged as a global epidemic and represents a major challenge with serious health implications (1). Given the well-established role of excess adiposity in increasing the risk of comorbid conditions and mortality (2, 3), obesity has been recognized as a leading health priority over the past 30 years (4).

Nutritional interventions are the cornerstone of obesity treatment and hold promise for personalized healthcare (5, 6). Among the nutritional approaches used for obesity management, the low carbohydrate diet (LCD) is a widely used and well-studied strategy for weight loss (7). LCD is defined as the consumption of less than 130 g of carbohydrates per day, which is equivalent to less than 26% of total energy from carbohydrates (8). It is recognized for its clinical benefits for individuals with obesity and has been extensively studied and reviewed by various associations and societies (9–12). A dose–response meta-analysis of 110 randomized controlled trials in adults with overweight or obesity found that every 10% reduction in carbohydrate intake was associated with a 0.64 kg greater weight loss within ≤6 months and a 1.15 kg greater loss at 12 months (13). The largest reductions in body weight occurred when carbohydrates provided approximately 30–35% of total energy intake, supporting carbohydrate restriction as an effective weight loss strategy in this population. However, individuals with obesity may experience variable weight loss responses to the LCD, reflecting the complex and multifactorial nature of obesity. Although LCD is widely used for weight management, the available evidence is still insufficient to fully explain the heterogeneity in weight loss outcomes (14, 71). In addition, many weight management studies focus on the average weight reduction at the group level, which can overlook clinically important differences in individual response, as some individuals achieve substantial benefit while others experience little or no improvement.

Integrating emerging technologies such as proteomics may help clarify the biological mechanisms through which LCD influences weight loss in individuals with obesity, and may also explain why responses to the same dietary intervention differ between individuals. Proteomics represent cutting-edge techniques employed to elucidate the complexities of biological systems (72). Within the domain of obesity, proteomics studies have been instrumental in identifying biomarkers and metabolic pathway alterations across different treatment modalities, including bariatric surgery (19–22, 73), anti-obesity medications (15), and nutritional interventions (21, 23–25). Despite this, literature on the plasma proteomics signature of LCD in individuals with obesity remains limited. This study aimed to characterize plasma proteomic changes observed during a 3-month LCD intervention in adults with obesity using an untargeted, exploratory proteomics approach and to identify candidate proteins associated with differences in weight loss response. In addition, we sought to compare proteomic signatures between individuals achieving clinically meaningful weight loss and those with limited weight loss response, in order to better understand the biological mechanisms underlying inter-individual variability. By integrating clinical and proteomic data, this study provides novel insights into the molecular adaptations to LCD and their potential implications for precision nutrition in obesity management.

## 2 Materials and methods

### 2.1 Ethical considerations and informed consent

The study protocol and procedures were approved by the Institutional Review Board, College of Medicine, King Saud University (no. E-23-7894), prior to undertaking the study. Written consents were obtained from all participants before their involvement in the study. The study was performed in accordance with the ethical standards of the Declaration of Helsinki and the universal International Conference on Harmonization-Good Clinical Practice Guidelines. Patients’ information was secured in the Obesity Research Center, College of Medicine, King Saud University.

### 2.2 Study design and participants

This is a three-month observational prospective cohort study, aimed at characterizing plasma proteomic profiles in patients with obesity who adhered to an LCD as part of their clinical treatment, using an untargeted approach. Patients were recruited from obesity clinics and the nutrition clinic at King Khalid University Hospital (KKUH) in Riyadh. The study included adult males and females with a BMI of ≥30 kg/m^2^, who were following up in the obesity or nutrition clinics and were instructed by their physician or dietitian to follow an LCD. Subjects were excluded if they were following an LCD before study enrollment; currently treated with anti-obesity medications; underwent bariatric surgery; diagnosed with end-stage renal disease or advanced liver decompensation; pregnant or breastfeeding women; or the presence of any condition that prevents the subject from participating based on the physician’s assessment. Before enrollment, dietary intake was assessed using two 24-h dietary recalls conducted on two non-consecutive days (one weekday and one weekend day) to confirm that participants were not already following an LCD. Eligibility required habitual carbohydrate intake above the LCD threshold (>120 g/day).

### 2.3 The low carbohydrate diet (LCD)

Participants were instructed by their dietitian to follow an LCD for 3 months (carbohydrates 60–110 g/day) as part of their clinical treatment. The dietary intervention aimed to reduce carbohydrate intake without intentional restriction of total energy intake. Participants were also instructed to maintain their habitual level of physical activity throughout the study period. Educational materials on the carbohydrate content of foods and drinks were provided, and participants were given direct access to the dietitian for dietary queries. Follow up was done regularly via phone calls and instant messaging to support adherence and monitor compliance.

### 2.4 Data collection

Anthropometric measurements were performed by trained research personnel. Weight and height were measured using calibrated devices, following a standard protocol in which the participants were wearing light clothing with no shoes on. Each variable was measured twice, and the average result was recorded. BMI calculated as weight/height^2^ (kg/m^2^). Waist and hip circumferences were measured using a flexible tape, and waist-to-hip ratio was calculated as waist/hip. Body composition assessment was performed using bioelectrical impedance analysis (InBody 970, Biospace, California, USA) by trained research personnel. At the end of the study, participants’ weight loss was assessed using percent total weight loss (%TWL), where a %TWL of ≥5% indicates clinically meaningful weight loss. The 5% threshold was selected based on the conventional definition of clinically meaningful weight loss (26, 27).

Dietary assessments were conducted using 24-h dietary recall on 2 non-consecutive days at baseline and at the end of the study period to ascertain a significant difference in carbohydrate intake between the two time points. The 24-h recall data was collected by a trained clinical dietitian utilizing the 5-step multiple-pass technique. During the dietary assessment, three-dimensional measurement aids (such as food models and household measures) and two-dimensional measurement aids (such as food photographs) were used to estimate portion sizes of consumed food. Carbohydrate content of participants’ intake was calculated to ensure that they adhered to the LCD.

Blood samples for proteomics studies were collected from each participant at baseline and at the end of the study, processed, stored, and analyzed according to the proteomics protocol, performed by trained technicians and phlebotomists. Twenty milliliters (20 mL) of fasting whole blood were collected by venipuncture and transferred into EDTA-coated tubes. Within 30 min of blood withdrawal, samples were centrifuged at 3,000 × g for 15 min to separate the plasma. After centrifugation, plasma was aliquoted and stored in multiple aliquots at −80 °C until analysis, in the biobank facility at Proteomics Unit, Obesity Research Center, College of Medicine, King Saud University.

Biochemical data, including fasting blood glucose, insulin, HbA1c, lipid profile, liver function tests, and renal function tests, were obtained from the participants’ medical charts. These laboratory measurements were conducted in the KKUH laboratory according to the hospital’s standard protocols using the automated cobas® 8,000 modular analyzer series (Roche Diagnostics GmbH, Mannheim, Germany).

#### 2.4.1 Protein extraction

Thawed plasma samples were depleted using PierceTM Top 12 Abundant Protein (Thermo Fisher Scientific, USA) for the high-abundance plasma proteins, according to the manufacturer’s instructions. Protein extraction was then carried out using TCA/acetone precipitation, as described by Chen et al. (28). Briefly, the depleted plasma samples were mixed (1:4 ratio) with ice-cold acetone containing 10% w/v TCA and vortexed for 15 s to ensure uniform mixing. The mixture was incubated overnight at −20 °C to precipitate the proteins. Following incubation, the samples were centrifuged at 1000 × g for 15 min at 4 °C, and the resulting pellets were solubilized in labeling buffer (7 M urea, 2 M thiourea, 30 mM Tris–HCl, 4% CHAPS, pH 8.5). The protein concentration of each sample was determined in triplicate using the 2D-Quantkit (GE Healthcare, Piscataway, NJ, USA). 50 μg of each sample was denatured in 6 M urea, reduced with dithiothreitol, alkylated with iodoacetamide, and digested overnight at 37 °C with 2.54 μg of modified porcine trypsin. The resulting tryptic peptides were desalted using Pierce C18 Spin Columns (Thermo Fisher Scientific) and subsequently dried using a speed vacuum centrifuge (29). Samples were processed simultaneously, and sample acquisition order was randomized to minimize potential run-order effects.

#### 2.4.2 Liquid chromatography coupled to tandem mass spectrometry (LC–MS/MS)

The digested peptides were dissolved in 0.1% (v/v) formic acid and then separated by a Dionex UltiMate 3,000 nano-LC system with a WPS-3000 autosampler. Peptides were loaded onto a trapping column (C18 PepMap100, 5 μm, 100 A) coupled to the analytical column (PepMap™ C18, 50 cm × 75 μm). Peptide separation was performed at a flow rate of 300 nL/min. Mobile Phase A consisted of 0.1% (v/v) formic acid in water, and Mobile Phase B consisted of 0.1% (v/v) formic acid and 80% (v/v) acetonitrile in water. The column was pre-equilibrated with 5% mobile phase B, followed by an increase to 22.5% mobile phase B over 139 min, then 45% mobile phase B over 184 min. After separation, the peptides were injected into the nanospray ion source for ionization and analyzed by a Q Exactive Plus Hybrid Quadrupole-Orbitrap mass spectrometer (Thermo Fisher Scientific, Waltham, MA, US) operating in positive ion mode, with a nanoelectrospray ionization (nESI) potential at 2000 V and a maximum duty cycle of 3 s. The primary mass spectrometry (MS) scan range was set to 375–1,650 m/z at a resolution of 70,000. For secondary mass spectrometry (MS/MS), the fixed first mass was set to 80 m/z, with a resolution of 17,500. The dynamic exclusion time was set to 20 s, and the automatic gain control was set to 3 × 10^6^ and 1 × 10^5^ for MS and MS/MS scans, respectively. Mass spectra were acquired in data-dependent mode (DDA).

#### 2.4.3 Proteomics data processing

Proteomics data analysis was performed utilizing Proteome Discoverer 1.3 (Thermo Fisher Scientific, USA), using Sequest as a search engine, and the HUMAN-refprot-isoforms.fasta as a sequence database. Raw spectra were first subjected to peak picking, followed by database searching using the following parameters: (1) pre-cursor/fragment mass tolerance: 15 ppm/0.02 Da; (2) a maximum of two missed cleavages; (3) trypsin (full) as the enzyme; (4) dynamic modifications of peptide N-terminal acetylation and methionine oxidation; and (5) static modification of cysteine carbamidomethylation. Peptide-spectrum matches (PSM) were then filtered, and the protein and peptide FDRs were set at 1%, with a minimum of two or more unique peptides per protein for protein identification. The filtered PSM list was exported and manually formatted in Excel. Only proteins identified and quantified via Label-free quantification (LFQ) intensity were used for subsequent analyses. The resulting list of quantified proteins, exported from Proteome Discoverer and manually formatted, underwent further non-redundant filtration (fold change ≥1.5 for upregulation and ≤0.67 for downregulation; *p* < 0.05; adjusted *p*-value (*q*-value) ≤0.01), and missing value removal. The final protein set was then subjected to multivariate statistical analysis using MetaboAnalyst v. 6.0,^1^ (McGill University, Montreal, QC, Canada) following median normalization, Pareto scaling, and log transformation (18). Principal component analysis (PCA) was performed for visualization of study groups and outlier detection, and partial least squares discriminant analysis (PLS-DA) was generated as a supervised model. Model performance was evaluated using fivefold cross-validation and 1,000 permutation tests. Heatmap and volcano plot analyzes were generated using MetaboAnalyst to visualize clustering patterns in the proteomic data and to highlight proteins showing significant differential expression between baseline and endpoint samples within each study group (15–17). Receiver operating characteristic (ROC) analyses were conducted to evaluate the ability of the selected significantly dysregulated proteins to discriminate between baseline and endpoint samples within each response group. These analyses were exploratory and were not intended to establish validated predictive biomarkers.

#### 2.4.4 Bioinformatics analysis

The list of the statistically significant dysregulated proteins was exported from the Proteome Discoverer then pathway analysis was carried out by Ingenuity Pathway Analysis (IPA) software.^2^ This software identifies the functions and pathways that are most strongly associated with the protein list by overlaying the experimental expression data on networks constructed from published interactions. The subcellular localization of all identified proteins was predicted by PANTHER (protein analysis through evolutionary relationships) classification system; a protein subcellular localization prediction tool.^3^ For the Gene Ontology (GO) enrichment analysis, proteins were classified by GO annotation into three categories, biological process, cellular compartment, and molecular function, according to the UniProt-GOA database.^4^

### 2.5 Statistical analysis

Anthropometric and biochemical data were checked for outliers and transfer errors. Normality was assessed for all continuous variables. Normally distributed data were presented as mean ± SD, while non-normally distributed data were presented as median (interquartile range). Paired samples *t*-test was used to compare normally distributed variables between baseline and endpoint, while Wilcoxon signed-rank test was used for non-normally distributed variables. The chi-square test was used to compare the distribution of categorical variables between responders and non-responders. All statistical analyses were done using the Statistical Package for Social Science (IBM SPSS®).

## 3 Results

### 3.1 Clinical and biochemical data

Thirty participants aged 18–50 years completed the study and successfully adhered to LCD, with carbohydrate consumption ranging from 60 to 110 grams per day. Dietary follow-up indicated that participants remained within this prescribed range throughout the study period. The baseline characteristics of the total study sample are presented in Supplementary Table S1. Overall, significant reductions were observed in body weight (−4.96 kg, *p*-value < 0.001), BMI (−2.3 kg/m^2^, *p*-value < 0.001), waist circumference (−4.1 cm, *p*-value < 0.001), hip circumference (−4.1 cm, *p*-value < 0.001), fat mass (−3.7 kg, *p*-value < 0.001), fat percentage (−1.4%, *p*-value < 0.001), gamma-glutamyl transferase (GGT) (−2.3 U/L, *p*-value = 0.034), triglycerides (−0.114 mmol/L, *p*-value = 0.033), and HbA1c (−0.11%, *p*-value < 0.001).

The magnitude of weight loss varied across participants; therefore, the study sample was further stratified based on weight loss response. Participants who achieved a total weight loss (%TWL) of ≥5% were considered to have a clinically significant weight loss and were classified as ‘responders’ (*n* = 15), whereas participants with %TWL of <5% were classified as ‘non-responders’ (*n* = 15). The two groups did not differ significantly in age or in most baseline characteristics; however, baseline hip circumference was significantly higher in responders than in non-responders (*p* = 0.024) (Table 1). Baseline muscle mass was higher in responders than in non-responders (30.24 ± 6.25 vs. 26 ± 5.79, *p* = 0.064); however, this difference did not reach statistical significance. Clinical and biochemical outcomes of the two groups at 3 months following LCD are presented in Table 2. Percent TWL achieved by each participant is displayed in Supplementary Figure S1.

**Table 1:** Baseline characteristics of the study sample stratified by weight response (%TWL).

Characteristics	Responders (%TWL ≥ 5) (*n* = 15)	Non-responders (%TWL < 5) (*n* = 15)	*p*-value
Age (years)	36.27 ± 10.02 (20–47)	34.47 ± 8.50 (18–50)	0.6
Sex			
Female	11 (73.4%)	13 (86.7)	0.651
Male	4 (26.6%)	2 (13.3%)	
Anthropometric measurements			
Weight (kg)	106.03 ± 14.76	94.82 ± 18.20	0.075
BMI (kg/m^2^)	41.24 ± 6.29	37.69 ± 5.42	0.109
Waist circumference (cm)	108.2 ± 13.79	104.5 ± 16.17	0.502
Hip circumference (cm)	134.03 ± 10.19	125.43 ± 9.52	**0.024**
Waist to hip ratio	0.81 ± 0.10	0.83 ± 0.08	0.574
Fat percent (%)	48.79 ± 5.38	49.72 ± 2.60	0.55
Fat mass (kg)	51.68 ± 8.88	47.17 ± 9.39	0.187
Muscle mass (kg)	30.24 ± 6.25	26 ± 5.79	0.064
Biochemistry			
ALT (U/L)	16.2 (12)	14.29 ± 6.23	0.111
AST (U/L)	15 (8.5)	14.55 ± 3.86	0.232
GGT (U/L)	20 (14)	19.2 ± 8.21	0.246
BUN (mmol/L)	3.71 ± 1.02	3.95 ± 1.38	0.582
Creatinine (µmol/L)	71.4 ± 1.02	65.47 ± 12.93	0.28
Total cholesterol (mmol/L)	4.8 ± 0.67	4.78 ± 0.91	0.93
HDL-C (mmol/L)	1.29 ± 0.23	1.3 ± 0.26	0.924
LDL-C (mmol/L)	2.96 ± 0.60	2.99 ± 0.86	0.9
Triglycerides (mmol/L)	1.21 ± 0.51	1.1 ± 0.32	0.327
HbA1c (%)	5.61 ± 0.29	5.52 ± 0.30	0.434
Fasting glucose (mmol/L)	5.44 ± 0.65	5.01 ± 0.64	0.76
HOMA-IR	5.02 ± 2.16	3.41 ± 2.52	0.7

Continuous variables were presented as mean ± SD if normally distributed, or as median (interquartile range) if not normally distributed. Categorical variables were presented as *n* (%). *p*-value < 0.05 is considered significant. ALT, alanine transaminase; AST, aspartate aminotransferase; GGT, gamma-glutamyl transferase; BUN, blood urea nitrogen; HDL-C, high-density lipoprotein cholesterol; LDL-C, low-density lipoprotein cholesterol; HbA1c, glycated hemoglobin; HOMA-IR, homeostatic model assessment of insulin resistance. Bold values indicate statistically significant *p*-values.

**Table 2:** Three-month LCD clinical and biochemical outcomes of the study sample stratified by weight response (%TWL).

Characteristics	Responders (%TWL ≥ 5) (*n* = 15)	*p*-value^*^	Effect size^**^	Non-responders (%TWL < 5) (*n* = 15)	*p*-value^*^	Effect size^**^
Anthropometric measurements						
Weight (kg)	98.27 ± 13.03	**<0.001**	2.136	92.65 ± 16.65	**0.002**	0.923
BMI (kg/m^2^)	37.49 ± 5.32	**0.003**	0.863	36.83 ± 4.85	**0.002**	0.952
%TWL	7.21 ± 2.37	–	–	2.61 (3.7)	–	–
TWL range	5.07–14.18	–	–	−2.26 to 4.74	–	–
Waist circumference (cm)	102.2 ± 12.27	**<0.001**	1.192	102.43 ± 14.14	0.110	0.417
Hip circumference (cm)	127.73 ± 10.41	**<0.001**	1.609	123.47 ± 9.95	0.045	0.537
Waist to hip ratio	0.8 ± 0.11	0.551	0.149	0.83 ± 0.09	0.946	0.017
Fat percent (%)	46.53 ± 6.43	**<0.001**	1.119	49.14 ± 2.84	0.088	0.447
Fat mass (kg)	45.77 ± 9.33	**<0.001**	1.717	45.61 ± 8.83	**0.004**	0.829
Muscle mass (kg)	29.31 ± 5.9	**<0.001**	1.725	25.73 ± 5.3	0.190	0.336
Biochemistry						
ALT (U/L)	20.5 (12.4)	0.532	0.161	14.42 ± 3.35	0.368	−0.025
AST (U/L)	15.8 (5.4)	0.842	0.051	15.58 ± 3.86	0.919	−0.227
GGT (U/L)	17 (14)	**0.004**	0.741	19.13 ± 8.81	0.930	0.022
BUN (mmol/L)	4 ± 1.22	0.248	−0.294	3.72 ± 0.85	0.533	0.156
Creatinine (µmol/L)	69.87 ± 17.02	0.249	0.293	64.6 ± 12.81	0.460	0.185
Total cholesterol (mmol/L)	4.87 ± 0.74	0.563	−0.145	4.87 ± 0.79	0.548	−0.150
HDL-C (mmol/L)	1.2 ± 0.23	0.2	0.328	1.33 ± 0.24	0.511	−0.165
LDL-C (mmol/L)	3.16 ± 0.74	0.07	−0.474	3.07 ± 0.67	0.570	−0.142
Triglycerides (mmol/L)	1 ± 0.4	**0.014**	0.683	1.03 ± 0.32	0.732	0.085
HbA1c (%)	5.52 ± 0.34	**0.006**	0.814	5.43 ± 0.32	**0.021**	0.636
Fasting glucose (mmol/L)	5.35 ± 0.7	0.526	0.159	5.24 ± 0.74	**0.024**	−0.616
HOMA-IR	3.91 ± 1.27	**0.004**	0.677	3.18 ± 1.44	0.681	0.102

Continuous variables were presented as mean ± SD if normally distributed, or as median (interquartile range) if not normally distributed. Categorical variables were presented as *n* (%). *p*-value < 0.05 is considered significant. ALT, alanine transaminase; AST, aspartate aminotransferase; GGT, gamma-glutamyl transferase; BUN, blood urea nitrogen; HDL-C, high-density lipoprotein cholesterol; LDL-C, low-density lipoprotein cholesterol; HbA1c, glycated hemoglobin; HOMA-IR, homeostatic model assessment of insulin resistance.

^*^Paired samples *t*-test for normally distributed variables and Wilcoxon signed-rank test for non-normally distributed variables.

^**^Hedges’ *g* for normally distributed data, and *r* for non-normally distributed data. Bold values indicate statistically significant *p*-values.

### 3.2 Proteomic analysis and identification

#### 3.2.1 Label-free quantitative proteomic analysis

Label-free quantitative proteomics was used to identify proteins at baseline and at the end of the study. In total, 716 and 779 nonredundant proteins were quantified based on two or more unique peptides in the responder and non-responder groups, respectively. Significant differentially expressed proteins were defined as those with a fold change ≥1.5 (upregulated) or ≤0.67 (downregulated) and a *p*-value < 0.05. Based on these criteria, 90 proteins (53 upregulated and 37 downregulated) were significantly differentially expressed between baseline and endpoint in the responder group, and 183 proteins (111 upregulated and 72 downregulated) in the non-responder group. Among these, 20 proteins were significantly altered in both groups (Supplementary Table S4).

#### 3.2.2 Analysis of differentially expressed proteins in response to LCD

**Figure 1:**
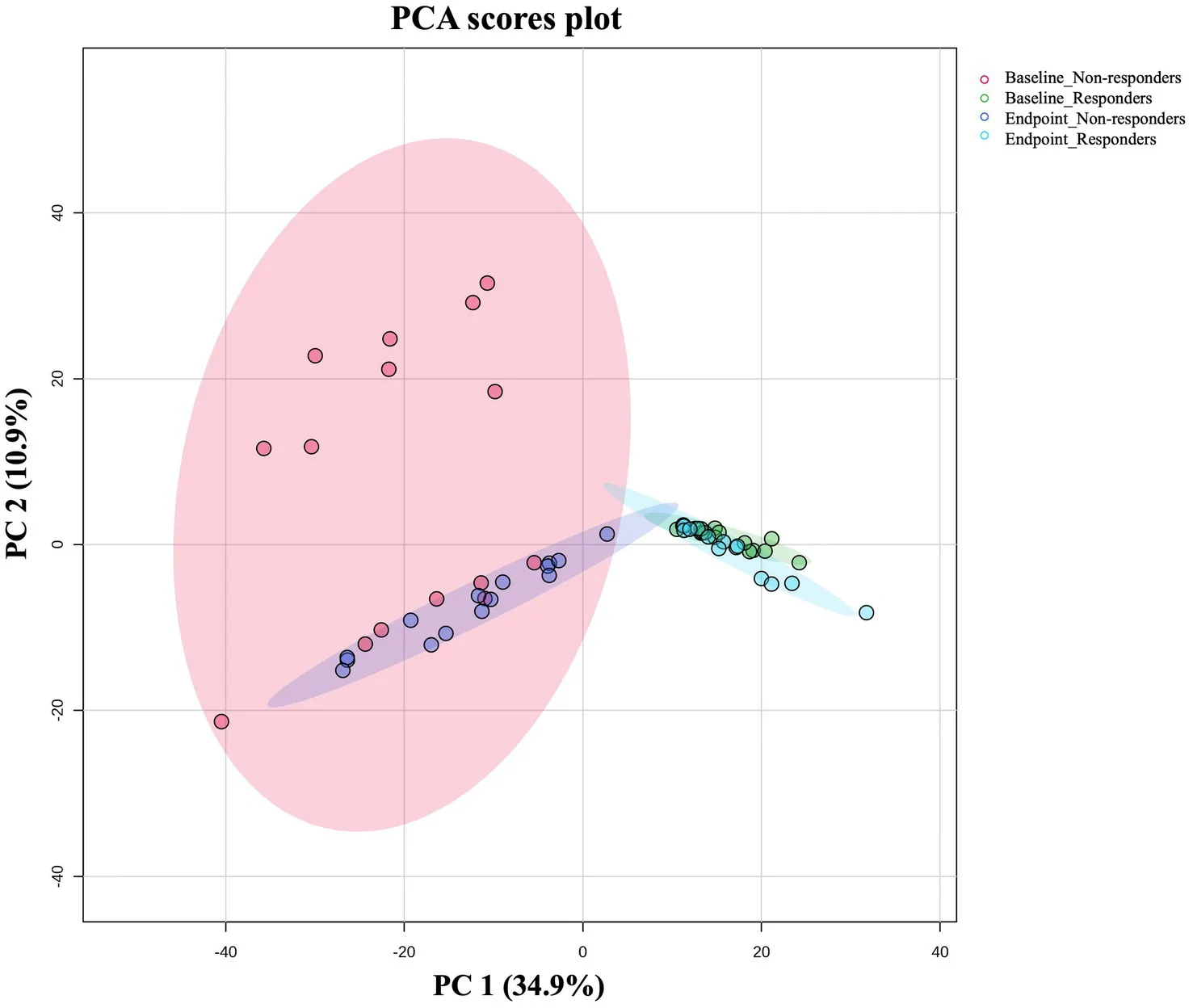
Multivariate analysis of plasma proteomic profiles of the responder and non-responder groups at the two time points (baseline vs. 3-month LCD). Two-dimensional principal component analysis (PCA) score plot showing a partial separation between the two timepoints in both groups.

**Figure 2:**
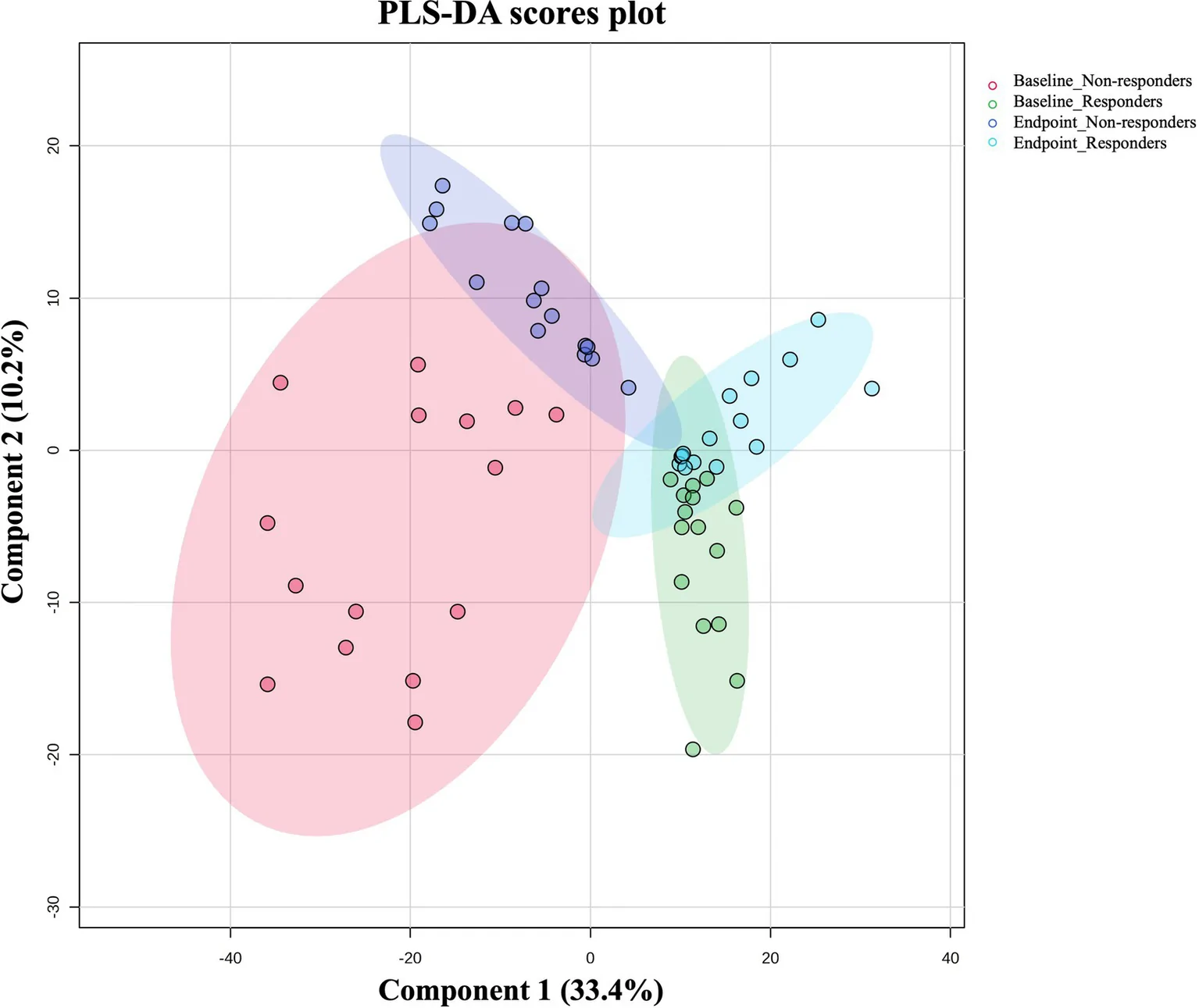
Partial least squares discriminant analysis (PLS-DA) score plot showing a partial separation between the baseline and endpoint plasma proteomic profiles in responders and non-responders. Model performance was evaluated using five-fold cross-validation and a 1,000-iteration permutation test.

Principal component analysis (PCA) was used to visualize group clustering and identify outliers. Figures 1, 2 showed the proteins that differentiated between the baseline and endpoint in the responder and non-responder groups. The PCA score plot showed partial separation between baseline and endpoint proteomic profiles in both groups (Figure 1). Partial least squares discriminant analysis (PLS-DA) showed separation among baseline and endpoint proteomic profiles in responders and non-responders (Figure 2). Five-fold cross-validation indicated increasing model performance across the evaluated components, with the five-component model achieving an accuracy of 88.3%, an *R*^2^ of 0.883, and a *Q*^2^ of 0.597. A 1,000-iteration permutation test showed that the observed classification performance was significantly greater than that obtained with randomly permuted class labels (empirical *p* < 0.001). These results support non-random separation of the study groups, although the model should be interpreted as exploratory because of the small sample size.

**Figure 3:**
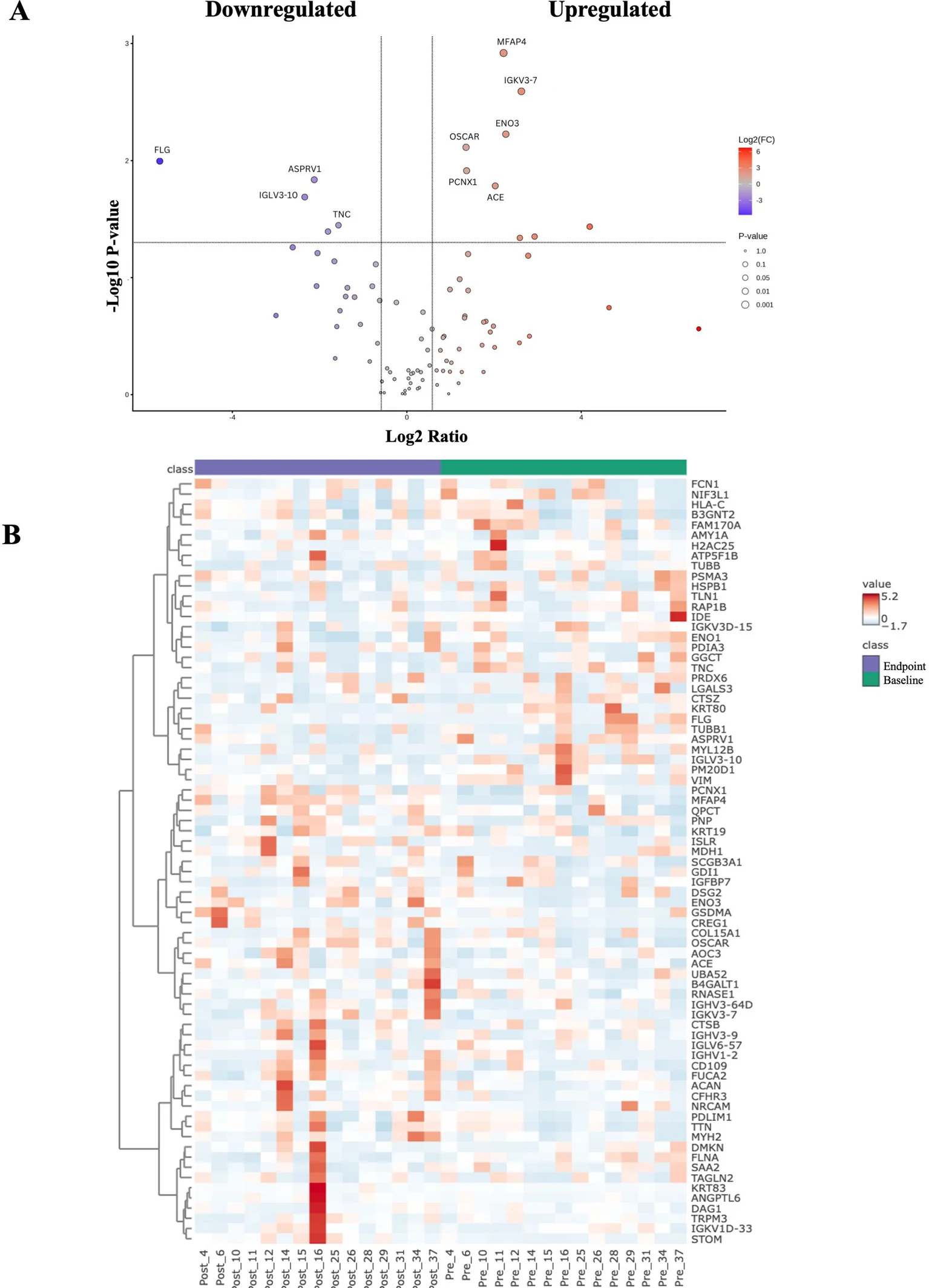
**(A)** Volcano plot showing a significant change in the levels of several proteins, of which red represents upregulated and blue represents downregulated plasma proteins in the responder group by the end of the study (FDR *p*-value <0.05-fold change ≥1.5). The vertical lines represent the fold change thresholds, and the horizontal line represents the statistical significance threshold. **(B)** Hierarchical clustering (HAC) and heatmap analysis of identified proteins that were significantly altered within the responder group (baseline vs. 3-month LCD). The color range bar indicates upregulated proteins as red and downregulated proteins as blue.

**Figure 4:**
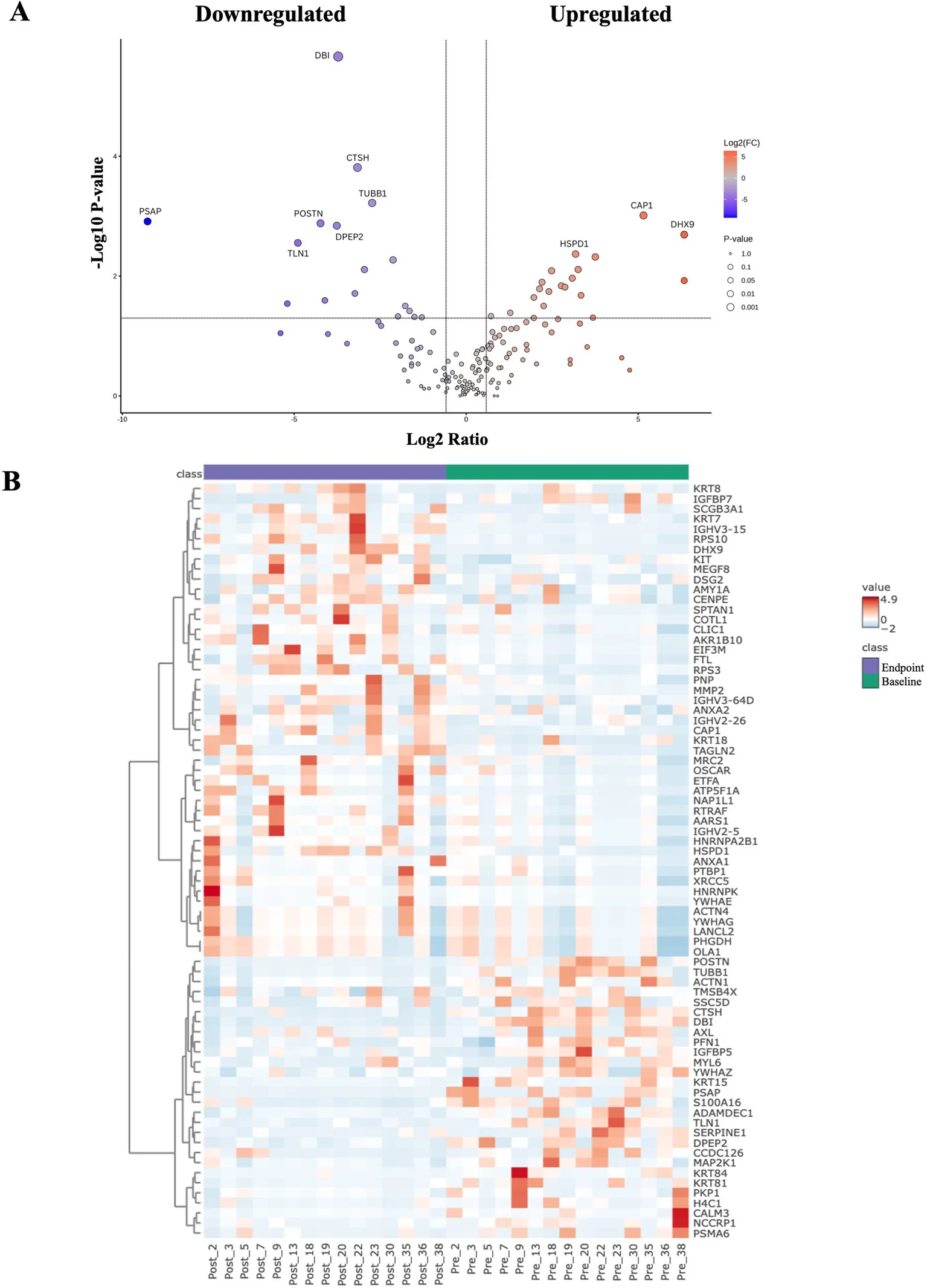
**(A)** Volcano plot showing a significant change in the levels of several proteins, of which red represents upregulated, and blue represents downregulated plasma proteins in the non-responder group by the end of the study (FDR *p*-value < 0.05-fold change ≥1.5). The vertical lines represent the fold change thresholds, and the horizontal line represents the statistical significance threshold. **(B)** Hierarchical clustering (HAC) and heatmap analysis of identified proteins that were significantly altered within the non-responder group (baseline vs. 3-month LCD). The color range bar indicates upregulated proteins as red and downregulated proteins as blue.

A moderated *t*-test (*p* < 0.05) with a fold-change cutoff of 1.5 was used to generate the volcano plots comparing baseline and endpoint samples in both groups. In the responder group, 90 proteins were differentially expressed, of which 53 (red) were upregulated and 37 (blue) were downregulated at the endpoint relative to baseline (Figure 3A). In contrast, the non-responder group showed 183 differentially expressed proteins, including 111 (red) upregulated and 72 (blue) downregulated proteins (Figure 4A). Differences in protein abundance are presented in the heatmaps in Figures 3B, 4B for the responder and non-responder groups, respectively. These differentially expressed proteins may serve as candidate biomarkers of the molecular and cellular changes associated with the LCD.

#### 3.2.3 Evaluation of significantly dysregulated proteins in the responder group

**Figure 5:**
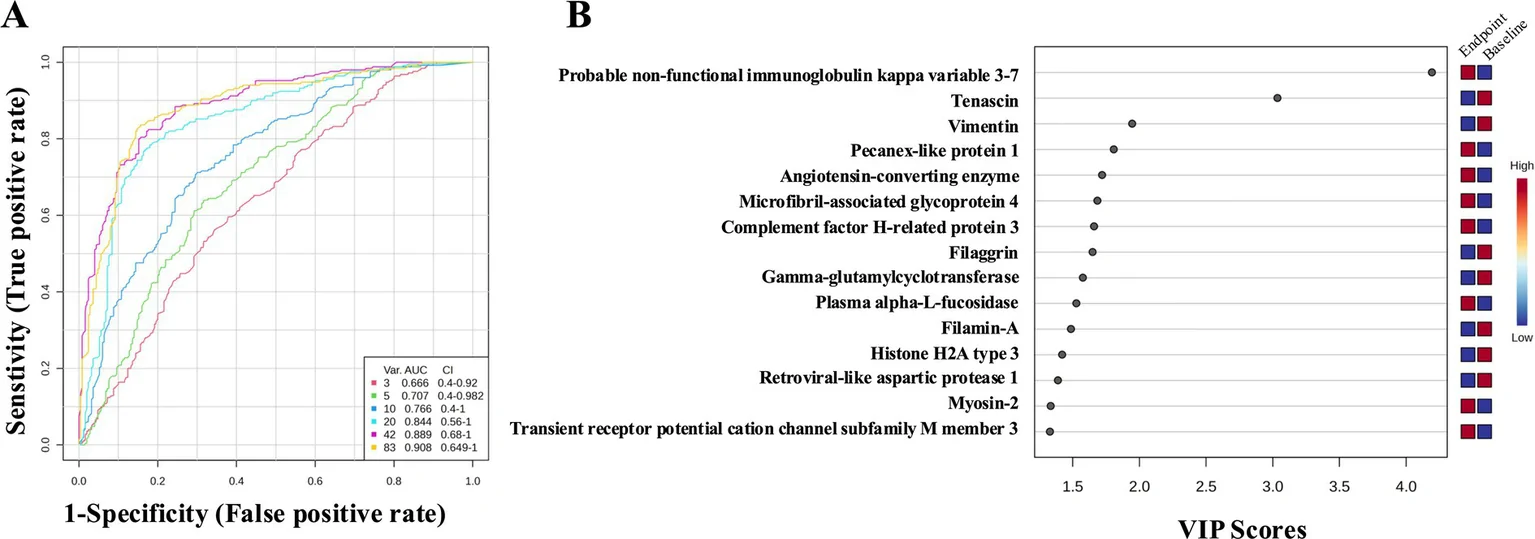
Evaluation of the significantly dysregulated proteins within the responder group (baseline vs. 3-month LCD). **(A)** Receiver operating characteristic (ROC) curve generated by the PLS-DA model, with area under the curve (AUC) values calculated from the combination of 3, 5, 10, 20, 42, and 83 proteins. **(B)** Variable importance in projection (VIP) score plot showing the 15 significantly dysregulated identified protein in the responder group pre and post LCD.

Significantly dysregulated proteins were further evaluated using receiver operating characteristic (ROC) curves. ROC analysis demonstrated good discriminatory performance of the multivariate proteomic model, with AUC values ranging from 0.666 to 0.908 (Figure 5A). Variable importance in projection (VIP) analysis identified the top 15 significantly dysregulated proteins between baseline and endpoint (Figure 5B).

**Figure 6:**
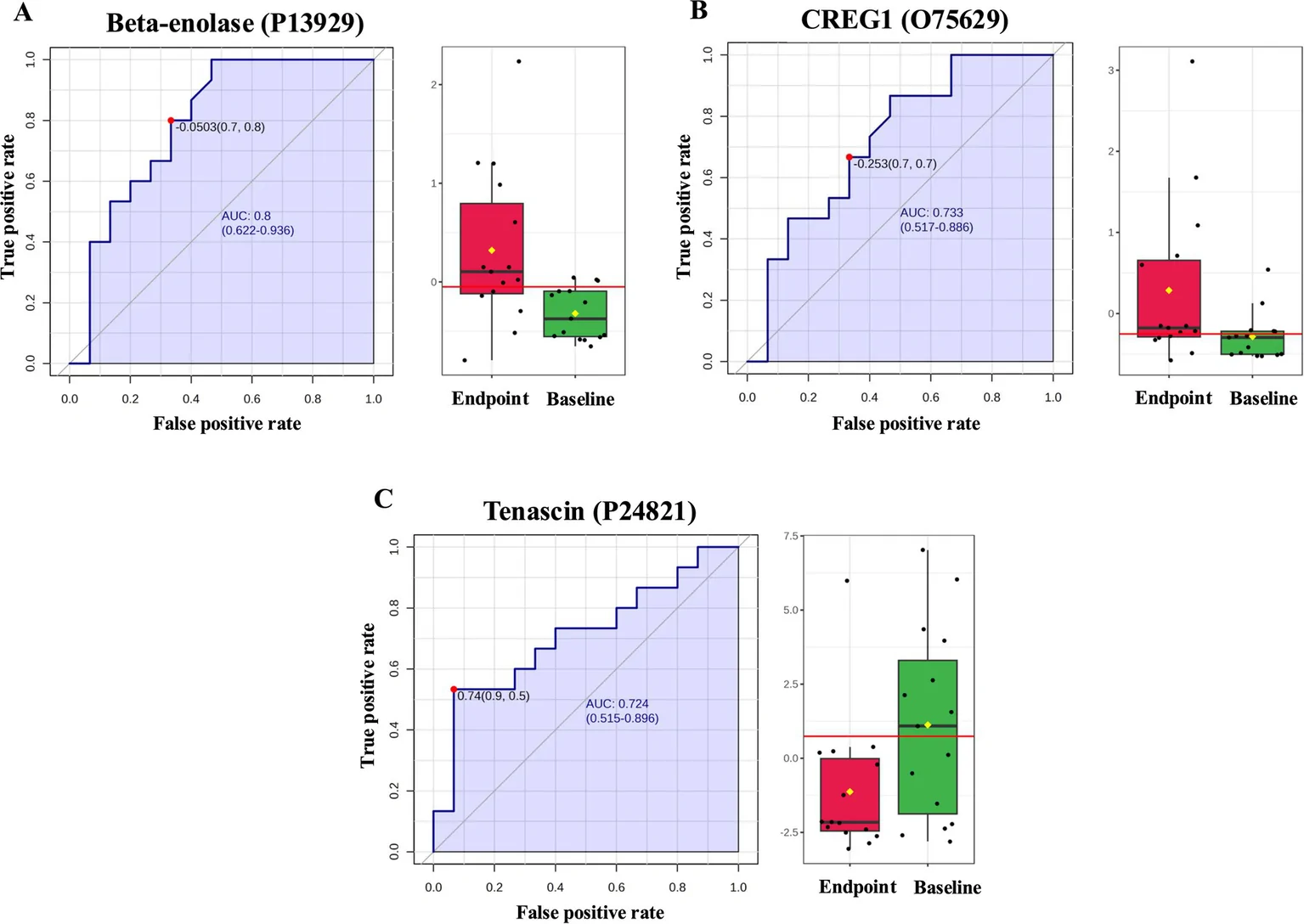
Receiver operating characteristic (ROC) curves and corresponding box-and-whisker plots are shown for selected proteins that were differentially expressed between baseline and endpoint in the responder group. Proteins were selected based on statistical significance and effect size (FDR-adjusted *p* ≤ 0.05 and fold change ≥1.5). Panels A and B show upregulated proteins: **(A)** Beta-enolase (P13929; AUC = 0.800), **(B)** CREG1 (O75629; AUC = 0.733), and panel C shows a representative downregulated protein: Tenascin (P24821; AUC = 0.724).

At the individual protein level, the AUC was 0.8 for beta-enolase (ENO3) (P13929) (Figure 6A), 0.733 for CREG1 (O75629) (Figure 6B), both were upregulated. The box-and-whisker plots showed increased expression of the two proteins at the endpoint compared with baseline. The AUC for tenascin (P24821) was 0.724 (Figure 6C), and the corresponding box-and-whisker plot showed decreased expression at the endpoint than at baseline.

#### 3.2.4 Evaluation of significantly dysregulated proteins in the non-responder group

**Figure 7:**
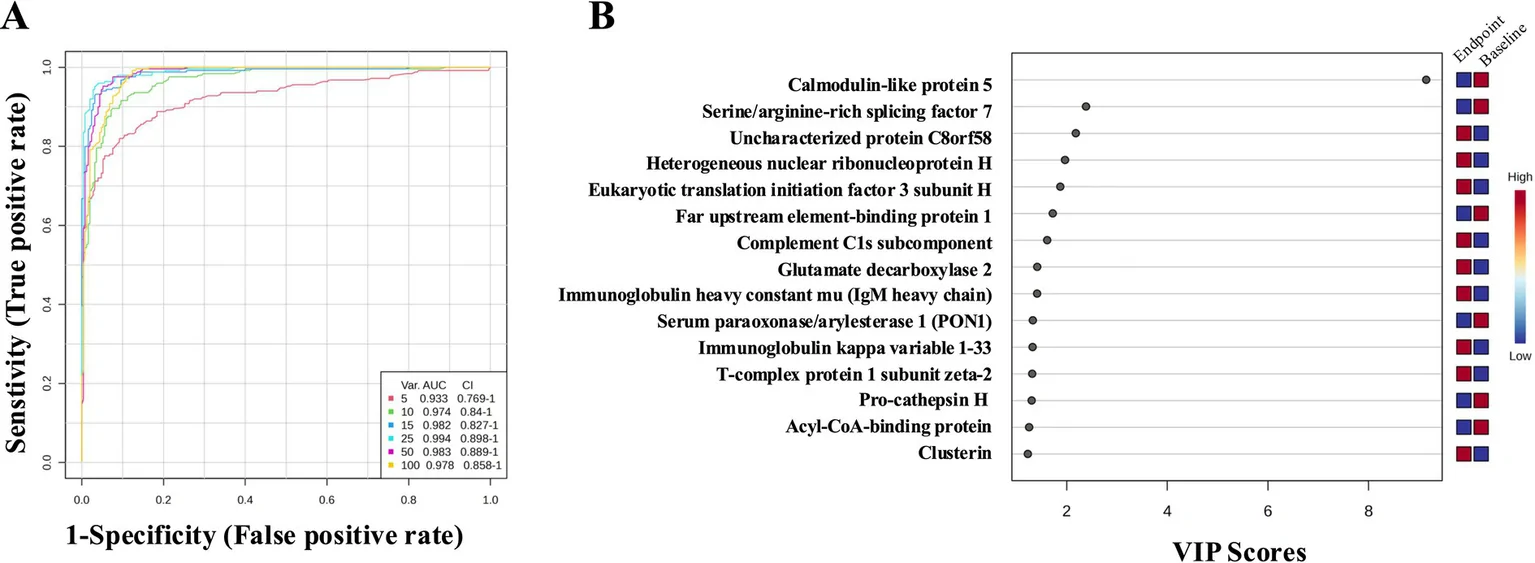
Evaluation of the significantly dysregulated proteins within the non-responder group (baseline vs. 3-month LCD). **(A)** Receiver operating characteristic (ROC) curve generated by the PLS-DA model, with area under the curve (AUC) value calculated from the combination of 5, 10, 15, 25, 50, and 100 proteins. **(B)** Variable importance in projection (VIP) score plot showing the 15 significantly dysregulated identified proteins in the non-responder group pre and post LCD.

ROC analysis demonstrated strong classification performance for distinguishing baseline and endpoint samples in the non-responder group, with AUC values ranging from 0.933 to 0.994 across models (Figure 7A). VIP analysis showed top 15 significantly dysregulated proteins between baseline and endpoint (Figure 7B).

**Figure 8:**
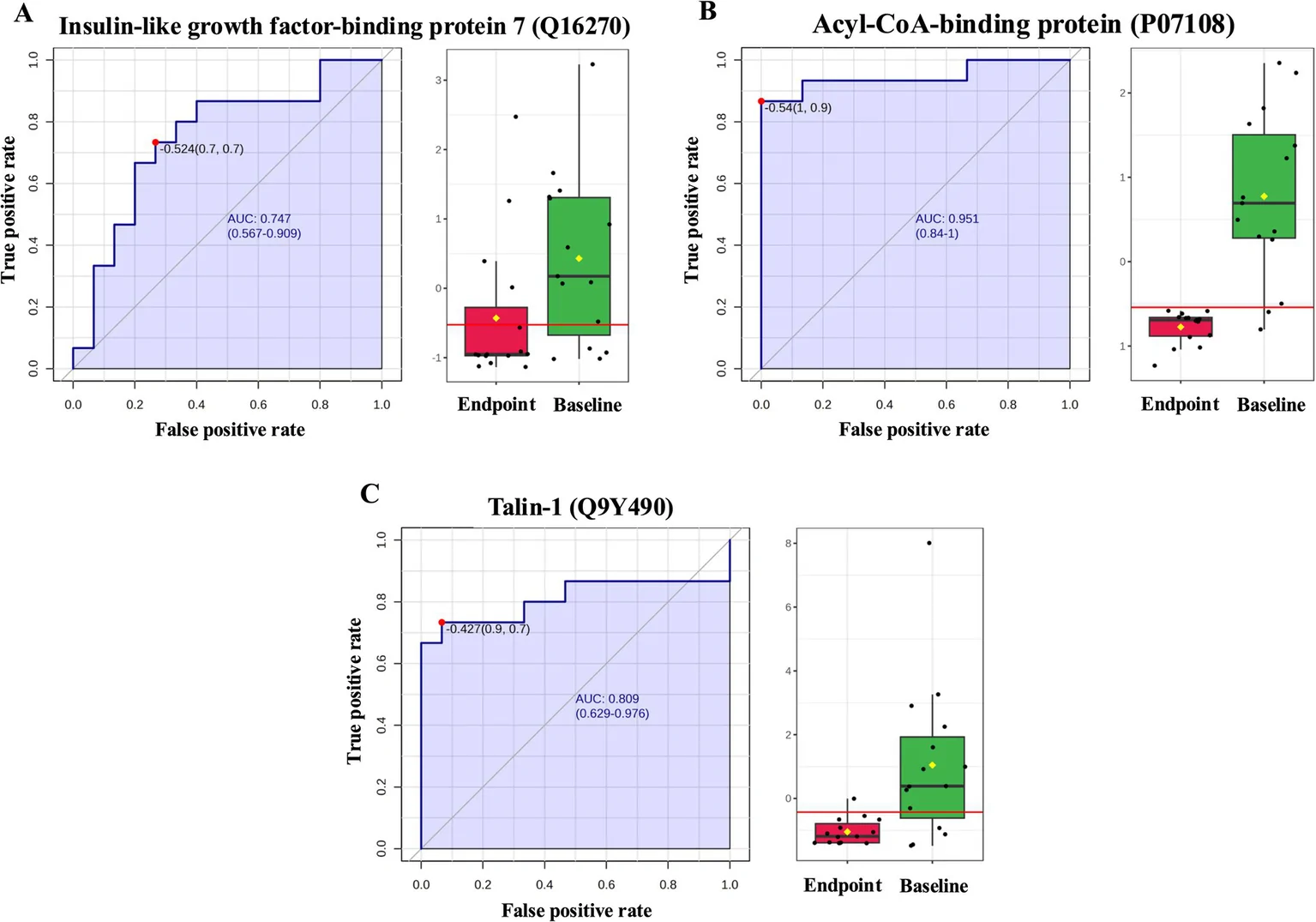
Receiver operating characteristic (ROC) curves and corresponding box-and-whisker plots are shown for selected proteins that were differentially expressed between baseline and endpoint in the non-responder group. Proteins were selected based on statistical significance and effect size (FDR-adjusted *p* ≤ 0.05 and fold change ≥1.5). Panels **(A–C)** show downregulated proteins: **(A)** Insulin-like growth factor-binding protein 7 (Q16270; AUC = 0.747), **(B)** Acyl-CoA-binding protein (P07108; AUC = 0.951), and **(C)** Talin-1 (Q9Y490; AUC = 0.809).

The AUC for insulin-like growth factor-binding protein 7 (IGFBP7) (Q16270) was 0.747 (Figure 8A), for acyl-CoA binding protein (ACBP) (P07108) was 0.951 (Figure 8B), and for talin-1 (Q9Y490) was 0.809 (Figure 8C); all were downregulated following the LCD. The box-and-whisker plots revealed lower expression of these proteins at the endpoint compared with baseline.

#### 3.2.5 Interaction network analysis of differentially expressed proteins

**Figure 9:**
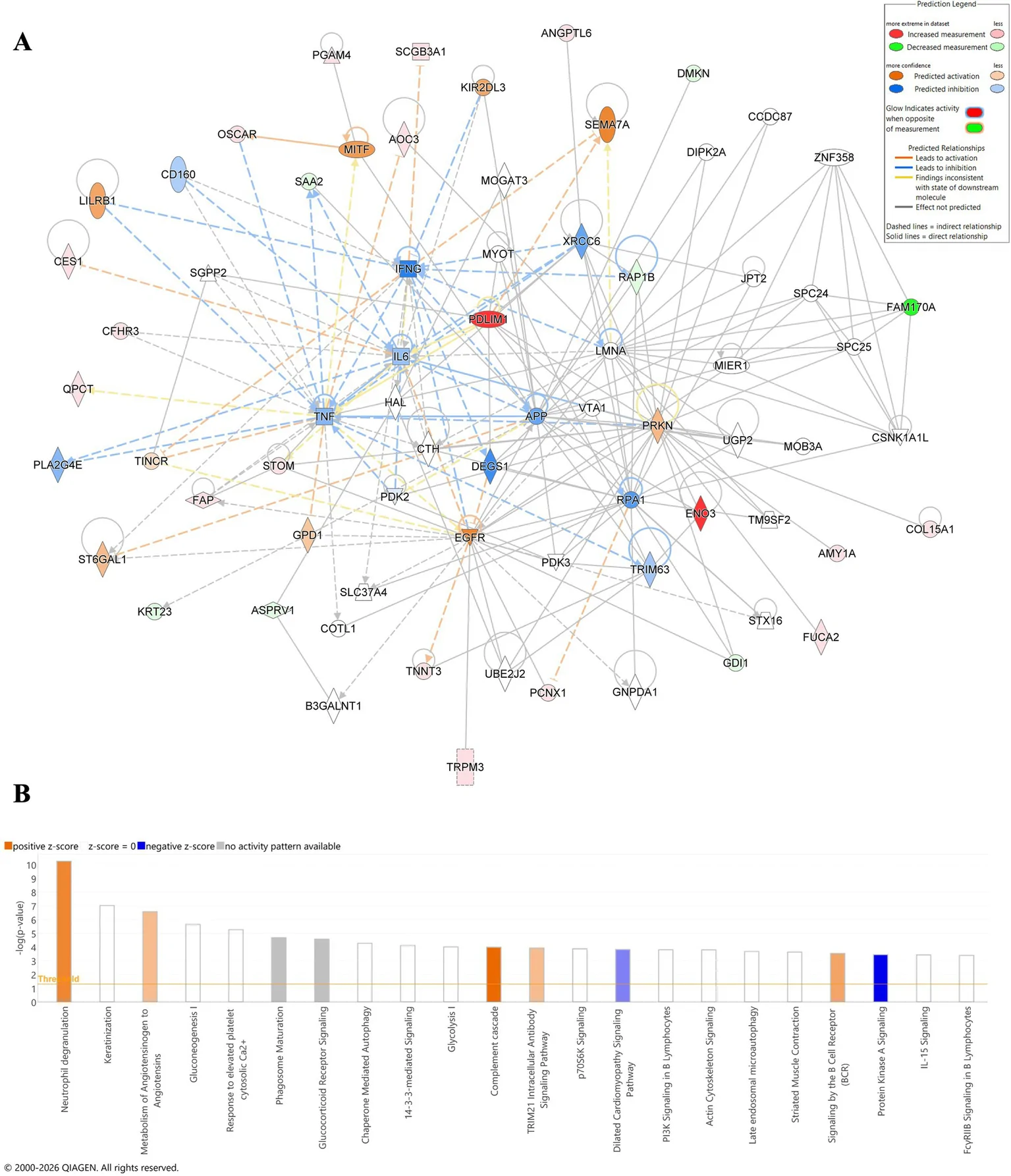
Schematic representation of the selected interaction network pathways depicting the involvement of the differentially regulated proteins in the responder group. **(A)** Protein interaction network pathway between the endpoint and baseline. Nodes colored green represent downregulation, and those colored red represent upregulation. The interaction networks were generated through IPA (QIAGEN, Inc.). **(B)** Top canonical pathways ranked by the *p*-values obtained by IPA. The blue bars represent negative *z* scores, the orange bars represent positive *z* scores, and the gray bars indicate that no activity pattern was available.

**Figure 10:**
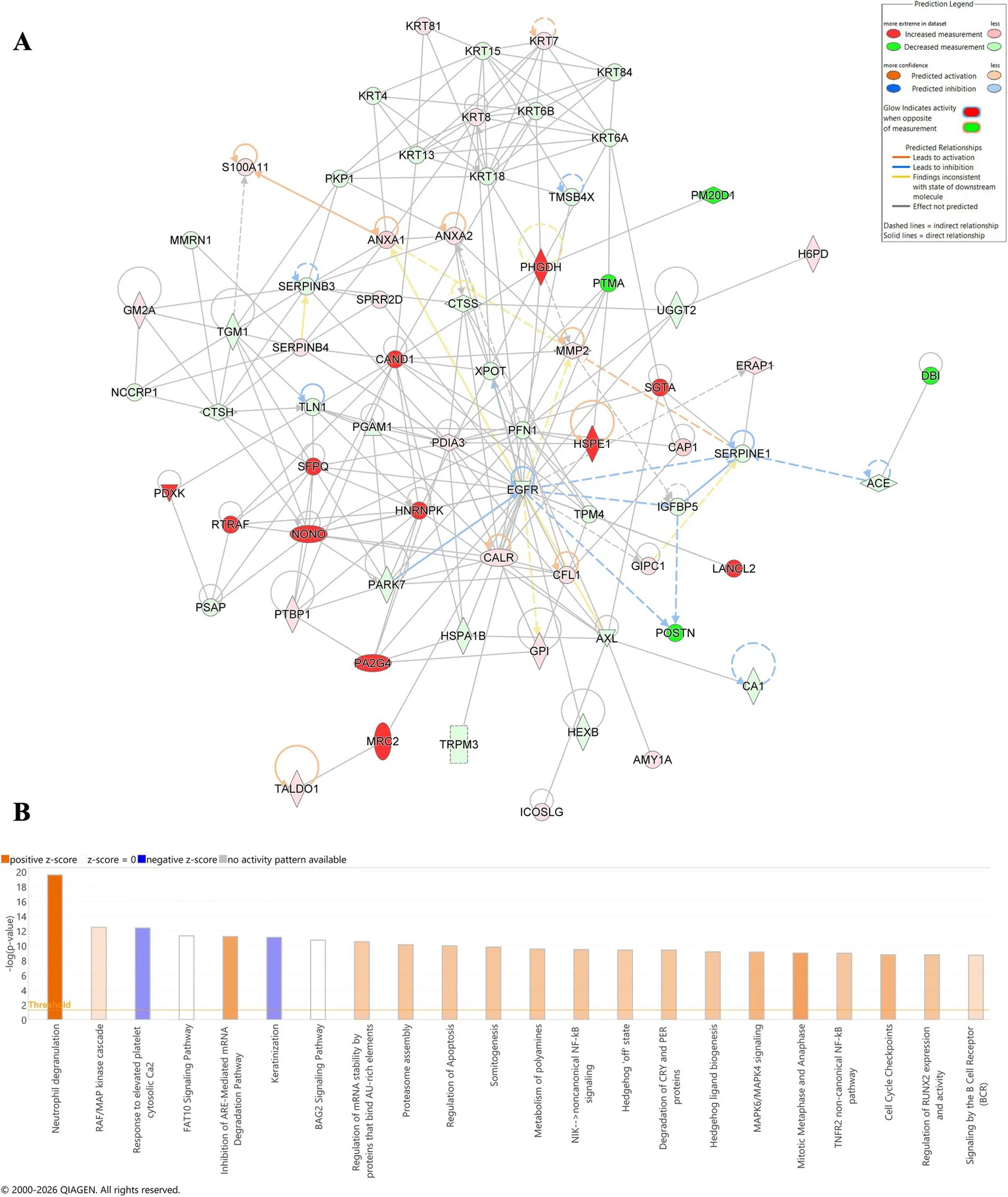
Schematic representation of the highest scoring network pathways depicting the involvement of the differentially regulated proteins in the non-responder group. **(A)** Protein interaction network pathway between the endpoint and baseline. Nodes colored green represent downregulation, and those colored red represent upregulation. The interaction networks were generated through IPA (QIAGEN, Inc.). **(B)** Top canonical pathways ranked by the *p*-values obtained by IPA. The blue bars represent negative *z* scores, the orange bars represent positive *z* scores, and the gray bars indicate that no activity pattern was available.

The biological relevance of the differentially expressed proteins was evaluated using Ingenuity Pathway Analysis (IPA). The software generates protein–protein interaction networks by mapping the input protein dataset to biological functions and interactions and calculates a score based on the best fit. The resulting networks are presented in Figures 9, 10.

In the responder group, the significant proteins mapped to three interaction networks. The second highest-scoring network showed the greatest biological relevance and was centered around the dysregulation of TNF, IL6, IFNG, APP, DEGS1, and ENO3 signaling pathways. This network was associated with cell-mediated immune response, cell-to-cell signaling and interaction, and cellular movement, with a network score of 43 and 25 focus proteins included (Figure 9A). Canonical pathway analysis showed significant enrichment of pathways related to neutrophil degranulation, metabolism of angiotensinogen to angiotensin, TRIM21 intracellular antibody signaling pathway, signaling by the B cell receptor, regulation of insulin-like growth factor transport and uptake by IGFBPs, among others, with a positive z score (Figure 9B).

In the non-responder group, significant proteins were related to four interaction networks. The highest-scoring network was centered around PGAM1, PDIA3, PHGDH, and EGFR, and was associated with cardiovascular disease, dermatological diseases and conditions, and organismal injury and abnormalities (Figure 10A). The network had a score of 154 and included 70 focus molecules. The canonical pathways associated with the altered proteins in the data set—including neutrophil degranulation, RAF/MAP kinase cascade, proteasome assembly, regulation of apoptosis, among others—were the most significant pathways with a positive z score (Figure 10B).

**Figure 11:**
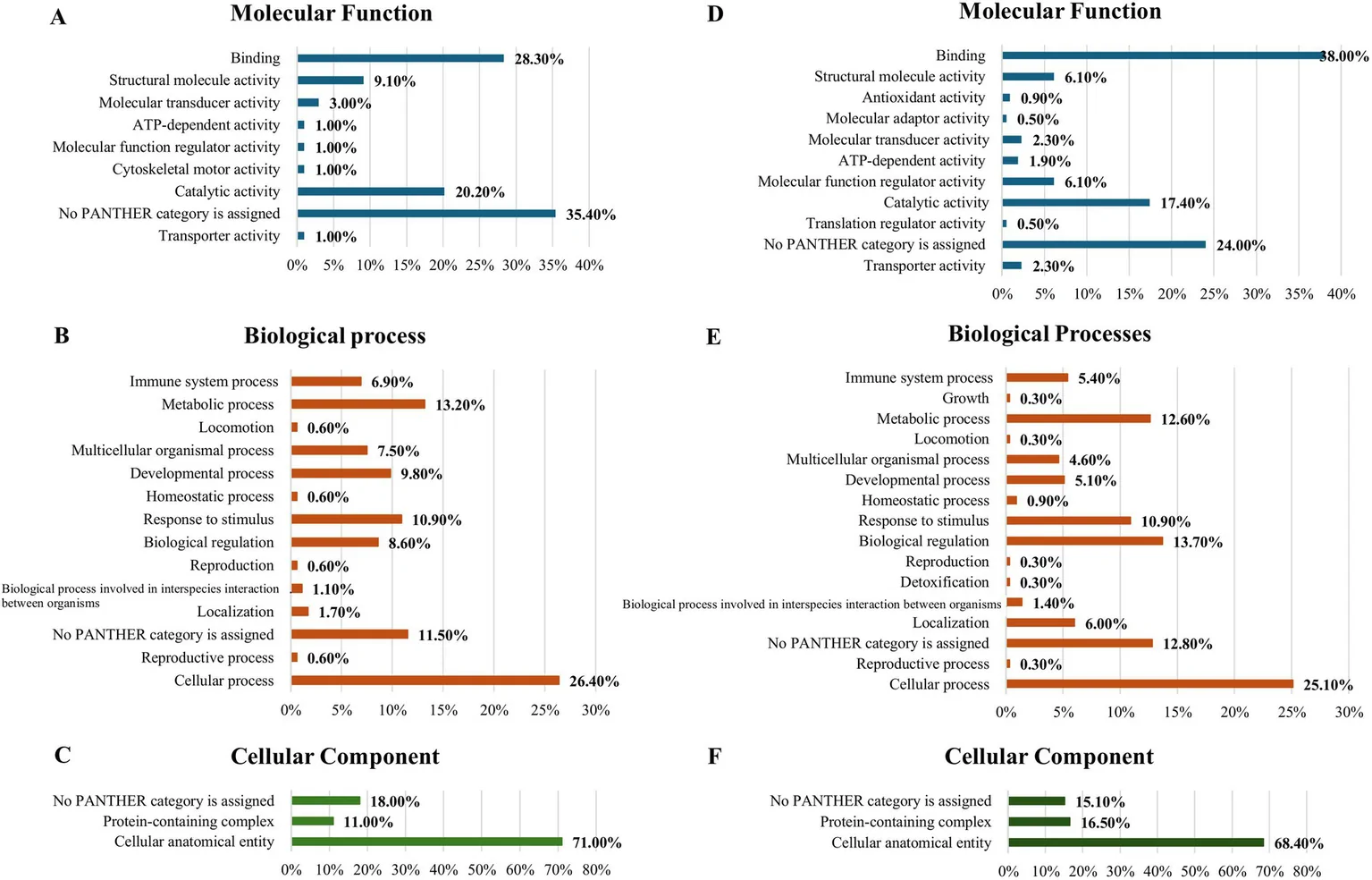
Comparative depiction of proteins identified in responders **(A–C)** and non-responders **(D–F)**, categorized according to their molecular functions, biological processes, and cellular components.

The protein analysis through evolutionary relationships (PANTHER) classification system was used to identify the proteins by their molecular functions, biological processes, and cellular components (Figure 11). In the responder group, most differentially expressed proteins were classified under enzymes with binding (28%) (Galectin-3, Talin-1, Dystroglycan 1, etc.), and catalytic activity (20.2%) (ENO1, ENO3, PDIA3, etc.) (Figure 11A). For biological processes, the identified proteins were mainly involved in cellular process (26.4%) (tenascin, microfibril-associated protein 4, macrophage mannose receptor 1, etc.) and metabolic process (13.2%) (PDIA3, ENO3, malate dehydrogenase cytoplasmic, etc.) (Figure 11B). Most proteins were located in the cellular anatomical entity (71%) (cathepsin B, ENO1, PDIA3, etc.), followed by the protein containing complex (11%) (dystroglycan 1, proteasome subunit alpha type-3, etc.) (Figure 11C). In the non-responder group, the most frequent molecular function categories were binding (38%) (insulin-like growth factor 1 (IGF1), alpha-actinin-1, annexin A1, etc.) and catalytic activity (17.4%) (EGFR, PHGDH, Angiotensin-converting enzyme, etc.) (Figure 11D), while the main biological process categories were cellular process (25.1%) (calreticulin, annexin A1, IGF1, etc.) and biological regulation (13.7%) (plasminogen activator inhibitor, 5′-AMP-activated protein kinase subunit gamma-2 (PRKAG2), IGF1, etc.) (Figure 11E). Most proteins were localized to cellular anatomical entity (68.4%) (annexin A1, IGF1, talin-1, etc.), followed by protein-containing complex (16.5%) (EGFR, 5’-AMP-activated protein kinase subunit gamma-2 (PRKAG2), Apolipoprotein A-V, etc.) (Figure 11F).

## 4 Discussion

In this prospective observational cohort study, we conducted untargeted label-free quantitative plasma proteomic profiling in adults with obesity to identify distinct molecular signatures observed following a 3-month LCD. Overall, study participants’ weight, BMI, fat mass, and HbA1c levels significantly decreased at the end of the study which falls in line with other major studies (30, 31). However, only half of the participants experienced clinically meaningful weight loss (TWL ≥ 5%). This study provides new insights into the molecular changes associated with the LCD by comparing distinct protein patterns between responders and non-responders. Because the study did not include a control diet group, these changes cannot be attributed exclusively to the LCD and should be interpreted as changes observed during the intervention period.

### 4.1 Candidate proteins and pathways associated with the biological response to LCD in the responder group

Responders showed an upregulation of ENO3, a catalytic enzyme located in the skeletal muscle tissue that plays a role in glucose catabolism and production (32). ENO3 converts 2-phosphoglycerate into phosphoenolpyruvate in the second to last step of glycolysis, and it functions in the reverse way during gluconeogenesis (33, 34). The upregulation of ENO3 may be consistent with altered energy metabolism and gluconeogenic adaptation during reduced carbohydrate intake. Nevertheless, given the absence of a control group, this protein change cannot be attributed specifically to carbohydrate restriction. A previous proteomic study of dietary weight loss interventions has also reported changes in proteins involved in metabolic regulation (23). However, ENO3 was not highlighted among the principal proteins identified in the study.

The protein CREG1 was upregulated in the responder group. Available evidence suggests that CREG1 is involved in obesity-related metabolic regulation and may play a protective role against obesity, insulin resistance, and hepatic steatosis; however, most of the studies are preclinical and have been conducted in animal models (35–37). Studies in mice have reported that increased expression of CREG1 stimulates brown/beige adipocyte formation, thereby ameliorating diet-induced obesity and its associated comorbidities such as fatty liver (38, 39).

On the other hand, tenascin was significantly downregulated in the responder group. Tenascin is an extra cellular matrix glycoprotein that is increased in subjects with obesity (40), and has been associated with chronic inflammation and metabolic stress (41). Continuous expression of tenascin was associated with several pathological conditions, including cancers, autoimmune, fibrotic, and metabolic diseases (42). Catalán et al. found that exogenous tenascin increased TLR4 and CCL2 expression in adipocytes, supporting a pro-inflammatory role in the adipose tissue (40). Therefore, the decrease in tenascin in responders may indicate the clinically meaningful weight loss observed during the LCD intervention was associated with attenuation of adipose inflammatory remodeling. Extracellular-matrix remodeling has also been observed in other dietary proteomic studies. Franzke et al. reported lower fibulin-1 and vitronectin levels after a high-protein diet, while Oller Moreno et al. identified proteoglycan 4 among the proteins showing the largest longitudinal changes during weight loss and maintenance (25, 74).

IPA analysis revealed a downregulation of TNF, IL6, IFNG, APP, and DEGS1, together with upregulation of ENO3. Overall, this pattern is consistent with a possible attenuation of obesity-related inflammatory signaling, as TNF, IL6, and IFNG are well-recognized mediators of adipose inflammation and insulin resistance (43–45), while APP has also been reported to be upregulated in adipose tissue in obesity and linked to adipose inflammation and insulin-resistant states (46, 47). Accordingly, the reduction of these nodes may reflect a shift toward a less inflamed metabolic environment. The reduced IL6-centered signaling in responders is consistent with Figarska et al., who found that greater BMI reduction was accompanied by larger decreases in circulating IL6 during low-fat and low-carbohydrate dietary interventions (23). Geyer et al. similarly reported improvement in a ten-protein inflammation panel following sustained diet-induced weight loss, while Oller Moreno et al. identified longitudinal changes in CRP, calprotectin, and serum amyloid A during weight loss and maintenance (24, 25). Hill et al. also found that reductions in refined grain intake were associated with reductions in the inflammatory proteins CD163 and IL1R-T1 during a behavioral weight loss intervention (69).

These findings may indicate a systemic transition toward improved metabolic homeostasis, where the reduction in inflammatory mediators parallels the observed improvements in anthropometric measures, body composition, glycemic parameters, and insulin resistance. Consequently, this proteomic signature may represent a molecular pattern associated with successful weight loss response during the LCD intervention rather than a direct effect attributable exclusively to carbohydrate restriction.

### 4.2 Candidate proteins and pathways associated with the biological response to LCD in the non-responder group

Insulin-like growth factor-binding protein 7 (IGFBP7) was downregulated in non-responders. It is a secreted insulin/IGF-binding protein involved in insulin signaling, vascular remodeling, and cellular senescence (75). Unlike the other IGFBP family members, IGFBP7 has relatively weak affinity for IGFs but a higher affinity for insulin (70), suggesting that it may interfere with insulin action and thereby contribute to insulin resistance (76). Moreover, it has been reported that higher IGFBP7 levels increased the risk of metabolic syndrome and insulin resistance (48). Previous studies have shown that increased plasma IGFBP7 level is positively associated with BMI and obesity (76), insulin resistance (76), metabolic syndrome (48), type 2 diabetes (49), coronary artery disease (50), and earlier obesity-related cancer diagnoses (51). Therefore, the observed downregulation of IGFBP7 in non-responders may represent a favorable shift, suggesting improvement in metabolic and vascular stress despite the absence of clinically meaningful weight loss. IGFBP7 was also among the proteins whose longitudinal changes were associated with changes in BMI in the DIETFITS proteomic substudy (23); however, that analysis combined participants assigned to the healthy low-fat and healthy low-carbohydrate diets and did not stratify them according to weight loss response.

Furthermore, non-responders showed significant downregulation of acyl-CoA-binding protein (ACBP). ACBP is a hunger-induced protein that stimulates appetite, increases food intake, and promotes lipogenesis, thereby contributing to obesity (52). Previous evidence has shown that circulating ACBP levels are elevated in individuals with obesity and decrease after substantial weight loss (52). Moreover, experimental studies have suggested that blocking ACBP can reduce obesity, diabetes, and fatty liver (53), further supporting its role as an obesogenic factor (54). In this context, the observed reduction in ACBP in the non-responder group may suggest that following an LCD led to improvements in appetite and lipogenesis-related signaling in individuals with obesity despite the absence of clinically significant weight loss.

Talin-1 was found to be significantly downregulated in the non-responders. Talin-1 is a key cytoplasmic protein that is essential for integrin activation and focal adhesion, and it is involved in cell–extracellular matrix adhesion, migration, apoptosis, and cytoskeletal remodeling (55, 56). Although the significance of talin-1 in obesity remains unclear, available evidence suggests that it is associated with related comorbidities. In plasma, higher talin-1 levels have been reported in patients with coronary artery disease and were associated with disease presence and severity, suggesting a potential role of talin-1 in the progression of coronary atherosclerosis (57). Moreover, increased serum talin-1 has also been associated with hepatocellular carcinoma, and a significant positive correlation between talin-1 and tumor size was reported, suggesting that it may serve as a prognostic marker for hepatocellular carcinoma (58, 59). Therefore, the downregulation of talin-1 in the non-responders after LCD may indicate attenuation of selected structural or vascular stress signals.

**Figure 12:**
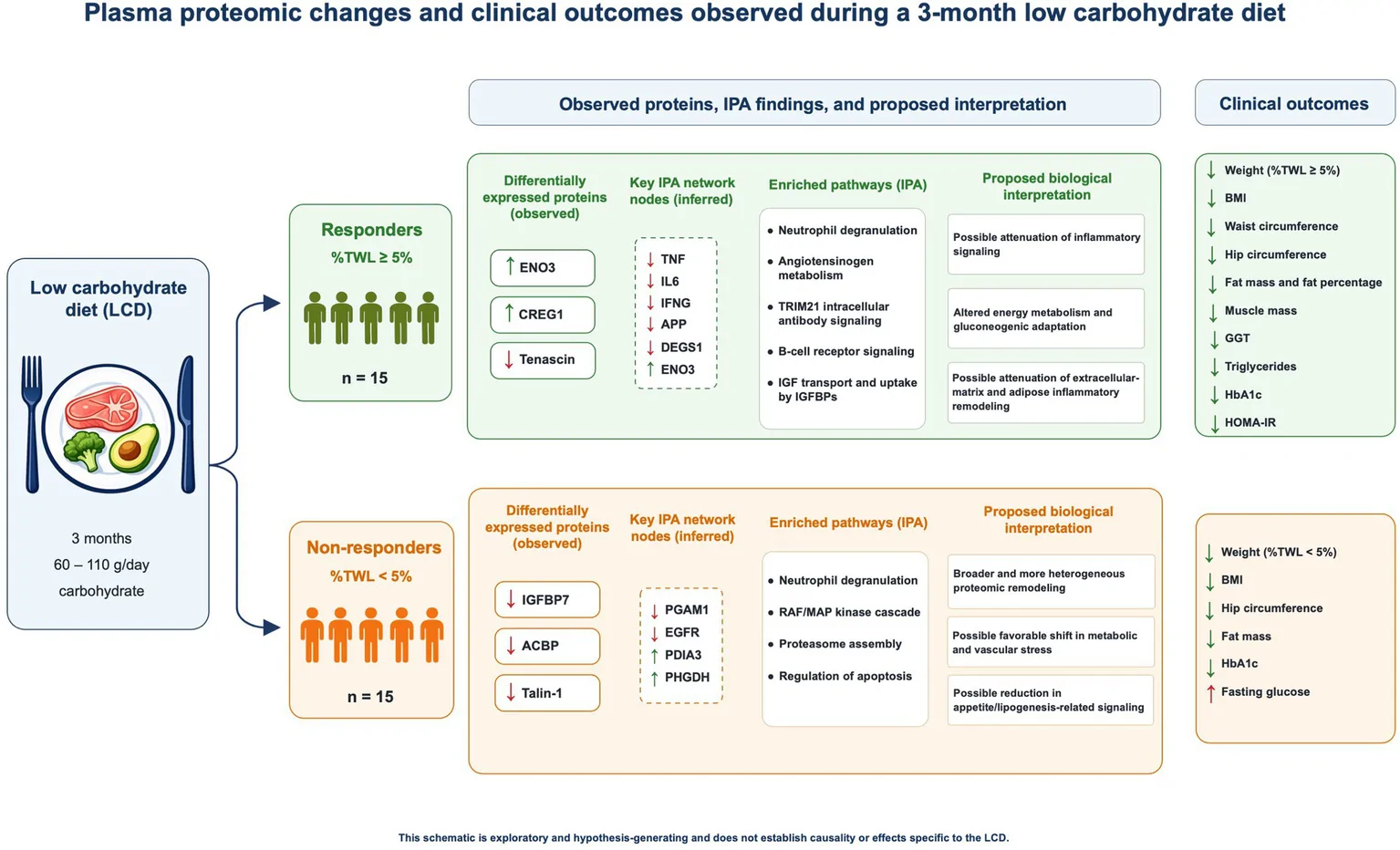
A schematic summary illustrating the proposed biological interpretation of plasma proteomic changes and clinical outcomes observed during a 3-month LCD intervention.

Compared with the responder group, the non-responders’ IPA network appeared to reflect a broader and more heterogeneous pattern of proteomic remodeling. IPA analysis showed a downregulation of PGAM1 and EGFR, and an upregulation in PDIA3 and PHGDH post LCD in non-responders. PGAM1 is a key glycolytic enzyme that is involved in adipose metabolic remodeling (60), suggesting that its downregulation may indicate altered glycolytic handling rather than a straightforward improvement or deterioration. Adipose tissue in subjects with obesity is characterized by a low-grade, chronic inflammation that leads to insulin resistance. Adipose tissue macrophages contribute to adipocyte dysfunction through the release of pro-inflammatory cytokines. Activation of EGFR has been linked with adipose dysfunction, and selective deletion of EGFR in mice decreased the development of insulin resistance and obesity in mice (61). In contrast, the upregulation of PDIA3 suggests persistent cellular stress, as PDIA3 is an endoplasmic reticulum stress-related chaperone whose circulating levels are elevated in obesity and positively associated with insulin resistance, dyslipidemia, and metabolic syndrome features (62, 63). PHGDH is a rate-limiting enzyme in serine biosynthesis and is linked to lipid and sphingolipid metabolism (64). Although adipocyte-specific PHGDH deletion has been reported to improve glucose intolerance in diet-induced obesity (65), hepatocyte-specific PHGDH deficiency leads to mild obesity and insulin resistance, indicating tissue-specific functions (66). In the present plasma-based study, increased PHGDH is perhaps best interpreted as evidence of ongoing metabolic adaptation rather than a clearly favorable or unfavorable alteration. The main plasma proteomic changes, associated pathways, proposed biological interpretations, and clinical outcomes observed in responders and non-responders are summarized in Figure 12.

Interestingly, baseline muscle mass was higher in responders than in non-responders. Although not statistically significant, this difference may have contributed to variation in weight loss outcomes through higher resting energy expenditure. Collectively, even in the absence of clinically meaningful weight loss, a 3-month LCD intervention facilitates meaningful improvements in body composition and glycemic markers, such as BMI, fat mass and HbA1c in non-responders. These clinical shifts suggest that the observed downregulation of pro-obesogenic and inflammatory proteins like ACBP and IGFBP7 represents an early molecular signature of metabolic improvement. Furthermore, the initiation of this proteomic remodeling suggests that extending the LCD intervention beyond 3 months may be necessary to translate these early molecular adaptations into significant phenotypic weight loss. Consequently, our findings indicate that proteomic remodeling may precede large-scale weight loss, offering a more sensitive metric for assessing the physiological benefits of dietary interventions in individuals who do not meet traditional weight loss thresholds.

### 4.3 Strengths and limitations

Key strengths of this study were its prospective design, paired baseline and endpoint assessments, and use of untargeted label-free quantitative proteomic approach, which allowed broad assessment of circulating protein changes during the LCD intervention. The established ≥5% total weight loss threshold also enabled a clinically relevant comparison of proteomic patterns between responders and non-responders. Standardized procedures were used for anthropometric measurements, blood collection, and sample processing. Dietary adherence was closely monitored throughout the study through regular follow-up with the clinical dietitian.

This study was limited by the relatively small sample size (*n* = 30), particularly for an exploratory proteomic analysis. This may have reduced the statistical power to detect modest but potentially meaningful protein changes, increased the possibility of false-negative findings, and limited the robustness and generalizability of the identified candidate proteins. The unequal proportion of male and female participants may also have introduced sex-related bias. Some measures were taken to minimize potential confounding. Participants were instructed to maintain their habitual physical activity, and dietary intake was monitored through regular dietitian follow-up. However, physical activity and medication use were not systematically assessed during follow-up, and some variability in dietary adherence and total energy intake may have occurred. Therefore, residual confounding from physical activity, energy intake, dietary adherence, and medication use cannot be completely excluded and may have contributed to the observed variation in weight loss and proteomic responses. Furthermore, the ROC analyses were conducted using the same cohort in which the candidate proteins were identified; consequently, the observed discriminatory performance may be overestimated and should be regarded as preliminary.

### 4.4 Future research directions and implications for precision nutrition

Several priorities emerge from this exploratory study. First, the candidate proteins identified in responders and non-responders should be validated in larger, independent, and sex-balanced cohorts. Because these proteins were identified using untargeted label-free proteomics, confirmation using targeted quantitative methods, such as ELISA, or targeted mass spectrometry, is required before they can be considered reliable biomarkers.

Future trials should also include an appropriate comparator diet, preferably with comparable energy intake, to distinguish proteomic changes associated with carbohydrate restriction from those related to weight loss or other nonspecific intervention effects. Repeated sampling at earlier and later time points, together with more objective assessment of physical activity and medication changes, would help clarify the clinical relevance of the observed proteomic changes. Longer follow-up is particularly important to determine whether the molecular changes observed in non-responders predict subsequent clinical improvement or reflect adaptations that occur independently of weight loss.

Another important direction is to investigate whether baseline protein profiles can prospectively predict response to an LCD. Future prediction models could combine proteomic markers with routine clinical characteristics and should undergo appropriate internal validation followed by testing in independent cohorts. Dietary trials would also be required to determine whether such biomarkers can identify individuals who are more likely to respond to an LCD than to another dietary approach. Integrating proteomics with metabolomics, lipidomics, gut microbiome composition, and genetic or epigenetic data may provide a more comprehensive representation of interindividual variability and support the development of more robust precision nutrition models (67, 68).

From a clinical perspective, a validated baseline biomarker panel could eventually help identify individuals who are more likely to achieve clinically meaningful weight loss during an LCD intervention and support earlier adjustment of dietary strategies. Serial biomarker assessment may complement conventional anthropometric and biochemical measures by identifying biological changes not captured by body weight alone. However, these exploratory findings are insufficient to guide diet selection, treatment duration, or clinical decisions. Clinical application will require validation in independent cohorts, standardized assays, and evidence that biomarker-guided dietary assignment improves outcomes beyond routine clinical measures.

## 5 Conclusion

In conclusion, this study identified distinct systemic proteomic changes observed during a 3-month LCD, characterized in responders by enhanced gluconeogenic adaptation (ENO3) and attenuated inflammatory signaling (TNF, IL6), and in non-responders by favorable reductions in metabolic and vascular stress markers (IGFBP7, ACBP, Talin-1) that corresponded with significant improvements in fat mass and HbA1c despite falling below the 5% weight loss threshold. Together, these findings highlight the sensitivity of plasma proteomics for detecting early physiological benefits of an LCD that may not yet be captured by anthropometric measures alone, and support its potential utility for biomarker discovery in precision nutrition.

## Data Availability

The original contributions presented in the study are publicly available. This data can be found here: MassIVE, MSV000101914.

## Footnotes

1 http://www.metaboanalyst.ca, accessed on 11 August 2025 and 23 February 2026.

2 Ingenuity Systems, http://www.ingenuity.com.

3 http://www.pantherdb.org.

4 http://www.ebi.ac.uk/GOA/.

## Supplementary material

SUPPLEMENTARY TABLE S1 Baseline characteristics of the total study sample (n = 30).

SUPPLEMENTARY TABLE S2 Proteomics results of the responder group.

SUPPLEMENTARY TABLE S3 Proteomics results of the non-responder group.

SUPPLEMENTARY TABLE S4 Common significant plasma proteins identified in both responders and non-responders, with direction of change in each group.

SUPPLEMENTARY FIGURE S1 A waterfall chart of percent total weight loss (%TWL) for each participant following a 3-month LCD.
