# Supplementary material for: The impact of low carbohydrate diet on the plasma proteomic profile of individuals with obesity
Source: Front Nutr. 2026 Sep 9;13:1856332. doi: 10.3389/fnut.2026.1856332 (PMC13599130; doi:10.3389/fnut.2026.1856332)
Supplement: Supplementary file 1 [file Supplementary_file_1.DOCX]

Supplementary Material

**Supplementary Figure 1:** A waterfall chart of percent total weight loss (%TWL) for each participant following a 3-month LCD.
