# Supplementary material for: The impact of low carbohydrate diet on the plasma proteomic profile of individuals with obesity
Source: Front Nutr. 2026 Sep 9;13:1856332. doi: 10.3389/fnut.2026.1856332 (PMC13599130; doi:10.3389/fnut.2026.1856332)
Supplement: Supplementary file 2 [file Table_1.DOCX]

Supplementary Material

**Supplementary Table S1.** Baseline characteristics of the total study sample (n=30).

| **Characteristics** | **Baseline** | **Endpoint** | ***P*-value^*^** | **Effect size^**^** |
| --- | --- | --- | --- | --- |
| **Age (years)** | 35.37 ± 9.2 (18-50)  (range: 18 - 50) | |  |  |
| **Gender** |  |  |  |  |
| Female | 24 (80 %)  6 (20 %) | |  |  |
| Male |  |  |  |  |
| **Anthropometric measurements** |  |  |  |  |
| Weight | 100.43 ± 17.25 | 95.46 ± 14.97 | < 0.001 | 1.204 |
| BMI (kg/m^2^) | 39.47 ± 6.04 | 37.16 ± 5.01 | < 0.001 | 0.689 |
| Waist circumference (cm) | 106.37 ± 14.89 | 102.32 ± 1.01 | < 0.001 | 0.778 |
| Hip circumference (cm) | 129.73 ± 10.63 | 125.60 ± 10.24 | < 0.001 | 0.969 |
| Waist to hip ratio | 0.82 ± 0.09 | 0.82 ± 0.10 | 0.631 | 0.086 |
| Fat percent (%) | 49.25 ± 4.18 | 47.84 ± 5.06 | < 0.001 | 0.771 |
| Fat mass (kg) | 49.42 ± 9.27 | 45.69 ± 8.92 | < 0.001 | 1.071 |
| Muscle mass (kg) | 28.12 ± 6.30 | 27.43 ± 5.77 | < 0.001 | 0.838 |
| **Biochemistry** |  |  |  |  |
| ALT (U/L) | 13.9 (7.45) | 15.75 (3.37) | 0.643 | 0.157 |
| AST (U/L) | 14 (10.95) | 14.5 (9.35) | 0.544 | 0.120 |
| GGT (U/L) | 18 (14.75) | 17 (14.25) | 0.022 | 0.594 |
| BUN | 3.83 ± 1.20 | 3.86 ± 1.04 | 0.882 | -0.027 |
| Creatinine | 68.43 ± 14.81 | 67.23 ± 15.04 | 0.165 | 0.253 |
| Total cholesterol (mmol/L) | 4.79 ± 0.79 | 4.87 ± 0.75 | 0.398 | -0.153 |
| HDL-C (mmol/L) | 1.29 ± 0.24 | 1.29 ± 0.23 | 0.917 | 0.019 |
| LDL-C (mmol/L) | 2.98 ± 0.73 | 3.12 ± 0.70 | 0.105 | -0.298 |
| Triglycerides (mmol/L) | 1.13 ± 0.43 | 1.02 ± 0.36 | 0.033 | 0.397 |
| HbA1c (%) | 5.56 ± 0.29 | 5.44 ± 0.29 | < 0.001 | 0.754 |
| Fasting glucose | 5.23 ± 0.67 | 5.30 ± 0.71 | 0.444 | -0.138 |
| HOMA-IR | 4.22 ± 2.45 | 3.54 ± 1.38 | 0.058 | 0.351 |

Continuous variables were presented as mean ± SD if normally distributed, or as median (interquartile range) if not normally distributed. Categorical variables were presented as *n* (%). *P* < 0.05 is considered significant. ALT, alanine transaminase; AST, aspartate aminotransferase; GGT, gamma-glutamyl transferase; BUN, blood urea nitrogen; HDL-C, high-density lipoprotein cholesterol; LDL-C, low-density lipoprotein cholesterol; HbA1c, glycated hemoglobin; HOMA-IR, homeostatic model assessment of insulin resistance.

^*^Paired t-test to compare the baseline and the endpoint.

^**^Hedges’ g for normally distributed data, and *r* for non-normally distributed data.
