# Supplementary material for: The impact of low carbohydrate diet on the plasma proteomic profile of individuals with obesity
Source: Front Nutr. 2026 Sep 9;13:1856332. doi: 10.3389/fnut.2026.1856332 (PMC13599130; doi:10.3389/fnut.2026.1856332)
Supplement: Supplementary file 3 [file Table_4.DOCX]

Supplementary Material

**Supplementary Table S4.** Common significant plasma proteins identified in both responders and non-responders, with direction of change in each group.

| **Protein name** | **Accession number** | **Responders** | **Non-responders** |
| --- | --- | --- | --- |
| MRC2 | Q9UBG0 | UP | UP |
| SCGB3A1 | Q96QR1 | UP | UP |
| IGHV3-64D | A0A0J9YX35 | UP | UP |
| OSCAR | Q8IYS5 | UP | UP |
| PM20D1 | Q6GTS8 | DOWN | DOWN |
| TRPM3 | Q9HCF6 | UP | DOWN |
| IGFBP7 | Q16270 | UP | DOWN |
| CFHR3 | Q02985 | UP | DOWN |
| AMY1A | P0DUB6 | UP | UP |
| DSG2 | Q14126 | UP | UP |
| ENPEP | Q07075 | UP | DOWN |
| KRT7 | P08729 | UP | UP |
| TAGLN2 | P37802 | DOWN | UP |
| TUBB1 | Q9H4B7 | DOWN | DOWN |
| TLN1 | Q9Y490 | DOWN | DOWN |
| H2AC25 | Q7L7L0 | DOWN | UP |
| PNP | P00491 | UP | UP |
| PDIA3 | P30101 | DOWN | UP |
| SAA2 | P0DJI9 | DOWN | DOWN |
| ACE | P12821 | UP | DOWN |
